# Pharmacist-directed vancomycin therapeutic drug monitoring in pediatric patients: a collaborative-practice model

**DOI:** 10.1186/s40545-021-00383-y

**Published:** 2021-11-30

**Authors:** Kashif Hussain, Rahila Ikram, Gul Ambreen, Muhammad Sohail Salat

**Affiliations:** 1 Department of Pharmacy, Aga Khan University Hospital, Aga Khan University Hospital, Stadium Road (Main Pharmacy), P.O Box 3500, Karachi, 74800 Pakistan; 2 Department of Pharmacology - Faculty of Pharmacy and Pharmaceutical Sciences, University of Karachi, Karachi, Pakistan; 3 Department of Paediatrics and Child Health, Aga Khan University Hospital, Karachi, Pakistan

**Keywords:** Therapeutic Drug Monitoring, Pharmacist, Vancomycin, Pediatric, TDM

## Abstract

**Background:**

Therapeutic drug monitoring (TDM) of Vancomycin (VCM) is required to prevent inappropriate dosage-associated bacterial resistance, therapeutic failure, and toxicities in pediatrics. Anecdotal experience and studies show that many healthcare institutions confront barriers while implementing TDM services, this study aimed to assess a pharmacist-directed VCM–TDM service for optimizing patient care in our institution.

**Materials and methods:**

Patients aged 1 month–18 years who received intravenous VCM were included in this quasi-experimental study. The pre-implementation phase (March–June 2018) consisted of retrospective assessment of pediatric patients, the interventional phase (July 2018 to February 2020) included educational programs and the post-implementation phase (March–June 2020) evaluated the participants based on pharmacist-directed VCM–TDM services as a collaborative-practice model including clinical and inpatient pharmacists to provide 24/7 TDM services. Outcomes of the study included the mean difference in the number of optimal (i) prescribed initial VCM doses (primary) (ii) dosage adjustments and (iii) VCM-sampling time (secondary). After ethical approval, data were collected retrospectively.

**Results:**

A hundred patients were there in each phase. The number of cases who were correctly prescribed initial VCM doses was significantly higher in the post-implementation phase, mean difference of 0.22, [95% CI (0.142–0.0.358), *p* < 0.0001]. Patients who had correct dosage adjustments in the post-implementation phase also had higher statistical significance, mean difference of 0.29, [95% CI (0.152–0.423), *p* < 0.05]. More correct practices of VCM-levels timing were observed in the post-implementation phase, mean difference of 0.15, [95% CI (− 0.053–0.264), *p* = 0.079].

**Conclusion:**

This study showed the significant role of pharmacist-directed TDM services to optimize the correct prescribing of initial VCM doses and dose adjustments.

## Introduction

Therapeutic drug monitoring (TDM) is the core responsibility of pharmacists for the delivery of optimum therapy to patients [1]. It is defined as the measure of blood concentrations of specific drugs at specified times for maintaining the concentration at steady-state and subsequently helps in individualizing the drug doses for the attainment of therapeutic targets [1, 2]. Aminoglycosides and vancomycin are the most monitored drugs especially in pediatric patients [3, 4]. Pharmacist-led TDM is reported to have positive patient outcomes including a reduced number of adverse drug effects (ADRs), shorten duration of therapy and hospital stay, lower rates of morbidity and mortality, and reduce the cost of therapy [1–3, 5–7]. Practically these patient-centered benefits can be achievable with the optimal utilization of pharmacists in healthcare settings [6, 8]. However, strong knowledge of the pharmacist about the pharmacokinetics (PK) principles and their practical implementation is required for effective TDM services [6].

In Pakistan, there is no published data about the assessment of hospital pharmacy TDM services and their effect on patient outcomes. However, a recently published Asian study reported that about 41% of hospitals in Riyadh, Saudi Arabia, engage their pharmacists in TDM activity and evaluate patient outcomes and recommend changes where needed [9]. The reported contribution of pharmacists is significantly lower than the rate reported in a 2010 survey by the American Society of Health-System Pharmacists (ASHP), where > 92% of American hospitals involved pharmacists for TDM [9, 10].

Medications PK monitoring-associated pharmacist responsibilities are established by ASHP [1]. Though, according to the current practices in our hospital pharmacists perform only a few of them, while working clinically and as an inpatient pharmacists. In the children’s units of the Aga Khan University Hospital, clinical pharmacists round with the physician team on weekdays in day shift, deliver TDM services, and routinely follow-up all the patients in pediatric and neonatal intensive care units (NICU and PICU), pediatric cardiac intensive care units (PCICU), and all pediatric special care and oncology units. In addition, pharmacist-based vancomycin TDM is provided to all admitted patients through extracting the data of all the patients on vancomycin therapy on daily basis by a dedicated pharmacist. Unlike other hospitals pharmacists in AKUH are not consulted for this activity but they are assigned to perform this task routinely [11]. All the newly hired pharmacists are trained for performing TDM activity during the training period and mentor-guided competencies of inpatient pharmacists are enhanced through educational lectures, supported by the provision of screening checklists and flowcharts [12] and finally evaluated before task assignments.

This study aimed to evaluate the impact of the collaborative-practice model, through implementing dedicated pharmacist directed-TDM services in combination/addition to the clinical pharmacist, and inpatient pharmacist to advance the vancomycin TDM and to overcome the barriers through the enhancement of pharmacist knowledge and skills regarding vancomycin TDM and providing vancomycin TDM focused 24/7 services for optimal patient care for hospitalized pediatric patients through the collaborative-practice model.

## Materials and methods

### Study design, settings, and duration

The impact of the implementation of pharmacist-directed vancomycin therapeutic drug monitoring as a collaborative-practice model on predefined outcome measures was evaluated in the pediatric units of Aga khan university hospital (AKUH), a teaching tertiary care hospital in Karachi, Pakistan, and associated with Aga Khan University. The study was performed after the approval of the institutional Ethical Review Committee (ERC) and the need for written informed consent was waived for retrospectively collected patients’ data.

We designed a single-center quasi-experimental study and compared 3 months (March–June 2018) of pre-implementation data with 3 months (March–June 2020) of post-implementation data. With implementation/training phase from July 2018 to February 2020. During this phase, all the standard operating procedures (SOPs) for pharmacist-directed VCM–TDM services were designed, and training was completed involving clinical, inpatients, and TDM-assigned pharmacists.

### Study population and sample size

In the present study pediatric patients of age > 30 days and less than 18 years were involved, who were admitted under the medical care in AKUH, either in the general ward or pediatric intensive care units (PICU & PCICU), and treated with intravenous VCM for suspected or confirmed infections during the specified study duration. All the pediatric patients admitted under the medical care were screened for inclusion, as ideally, all patients on VCM therapy need to be monitored for blood level. However, for all the pediatric cases who were exposed to multiple courses of VCM therapy during pre- and post-phases of study, only the first course of VCM exposure was included in data analysis. Patients who received only 24–72 h of empiric VCM therapy, were also included, as VCM trough levels are required before the 4th schedule dose at a steady state [13]. We excluded all the patients who started antibiotic therapy prior to hospital admission and information about the therapy start time, sample collection time, and trough values were missing. Patients who received VCM post cardiothoracic surgery, who had congenital heart diseases (CHD), congenital anomalies, and febrile neutropenia were also excluded. The **s**ample size was estimated of 100 participants in each group to give a 90% power to identify a difference of 21% in pre and post-intervention phases for the frequency of prescribing correct initial doses of vancomycin [7] at 95% CI using PASS version 11. Patients were included by convenience sampling from the list of all eligible cases provided by the Information Technology (IT) dept.

### Clinical and inpatient pharmacists’ activities

In AKUH, clinical pharmacists are performing clinical activities by attending daily ward rounds with the physician teams and recommend drug interventions for optimization of therapy with effective follow-ups to monitor the patients for the attainment of targeted therapeutic outcomes. In addition, under the direction of the manager of clinical pharmacy, clinical pharmacists perform a number of other drug-related responsibilities such as monitoring and reporting medication-safety through reporting adverse drug reactions (ADRs), participate in developing drug protocols, providing medication reconciliation and counseling, running antibiotic stewardship program, and participating as active members in the antibiotic subcommittee, etc. Clinical pharmacists are also contributing to clinical research and training pharmacy interns, new pharmacists, physicians, and nurses. Nevertheless, they are not assigned to verify the electronically prescribed physicians’ medication orders. Conversely, inpatient pharmacists assigned as in-charge in pharmacies are primarily responsible for electronic verification of physicians’ orders. In our hospital, physicians place medication orders through computerized physician order entry (CPOE) for all the hospitalized patients and these orders are verified and processed by the inpatient pharmacists through CPOE. They do not participate in daily ward rounds and are not responsible for patient follow-up for the achievement of desired therapeutic goals.

### Implementation of pharmacist-directed vancomycin TDM

The project comprised of Pre-pharmacist directed-TDM phase (pre-PD-TDM-phase), intervention-phase, and post-pharmacist directed-TDM phase (post-PD-TDM-phase). In the pre-PD-TDM-phase of 3 months, the study pharmacist assessed all the eligible patients retrospectively. In the intervention phase, the manager clinical pharmacy designed the VCM TDM educational sessions for all pharmacists, involved antibiotic subcommittee and P & TC members for the approval of this activity by pharmacists, and highlighted the importance of this prospective audit and interventional activity for the achievement of clinical outcomes. Then involved all the clinical and inpatient pharmacists and provided vancomycin PK-based interactive sessions in the period of about one and half years to cover all the on-board inpatients and clinical pharmacists. For the frontline pharmacist convenience and learning, pharmacists were equipped with age-based dosing and monitoring checklist and flowcharts of VCM regimens. P & TC approved AKUH antibiotic guidelines are developed in accordance with the ASHP/IDSA guidelines [14] and drug monographs. These guideline-based sessions were also included, and a pocket copy was also provided to each participant. Participants were assessed initially through assessment questions. Before and after each session’s test was conducted to assess the pharmacist's knowledge. Based on assessment results pharmacists were assigned vancomycin TDM activity. Role of clinical and inpatient pharmacist defined in this collaborative model under the direct supervision and direction of manager clinical pharmacy. Pharmacists were further assessed for the execution of their knowledge and skills during this period.

In the post-PD-TDM phase, the assigned TDM-pharmacist got fully involved in the provision of vancomycin–TDM services and daily applied a system-based filter to get the drug charts data of all the patients on VCM to review the appropriateness of therapy and drug levels ordering. This pharmacist communicates with the physician team through a call for the communication of dose optimization and level ordering. In addition, pharmacist directed-TDM services also involved the provision of information and recommendation to the primary care doctors’ team, infectious disease physicians, and clinical pharmacists about the vancomycin initial dosing regimen, dosing adjustments, and serum blood concentration requests. Clinical pharmacists also covered all the pediatric patients during clinical pharmacist rounds (including PICU, PCICU, pediatric special care, and oncology units) for TDM services. These both pharmacists were available during day shifts and on weekdays only. All the pending follow-ups for dose adjustments and levels ordering were carried out by the inpatient pharmacists, who give 24/7 service.

### Data collection

Data of all demographic and clinical variables were collected retrospectively. Date of the correctness of initial VCM doses, dose adjustments, ordering time, all the therapeutic interventions, communications, follow-ups, laboratory results, and approvals was maintained in an excel sheet and shared with clinical pharmacy manage on daily basis, who also facilitate the communication among all TDM-pharmacists, clinical and inpatient pharmacists around the schedule.

### Outcome measures

The primary outcome of the study was to assess the influence of pharmacist-directed TDM intervention on the frequency of prescribing the correct initial dose of vancomycin in pediatric patients as per institutional antibiotic guidelines by comparing pre- and post-intervention phases. This study had secondary outcomes including the evaluation of difference in the proportion of dosing correction/adjustment within 8 h (same pharmacist shift) and optimal blood sampling time practices. The drug levels orders were defined optimal if the sample drawing time for the initial trough levels of vancomycin is within 1 h before the scheduled time of the 4th dose administration [13].

### Statistical analysis

Statistical analysis was run using STATA version 15 (STATA Corp, Texas). The baseline characteristics of the study participants, orders of initial vancomycin dosages, dose adjustments, and blood sampling times were described and reported by descriptive statistics. We used Fischer’s exact and χ2 tests if found cells count of ≤ 5 for categorical and binary variables. The non-normally distributed continuous baseline variables were compared by Mann–Whitney test. A two-sample test of proportion was applied to evaluate proportions mean difference and 95% CI for the orders of VCM-initial dosages, dose adjustments, and blood sampling times. We used two-sided tests and a *p* value < 0.05 and 95% confidence interval (CI) were deemed statistically significant.

### Ethical approval

Before performing this study, ethical approval was taken from the institutional ethical committee of Aga Khan University Hospital (ERC # 2020-5150-11,679).

## Results

**Fig. 1:**
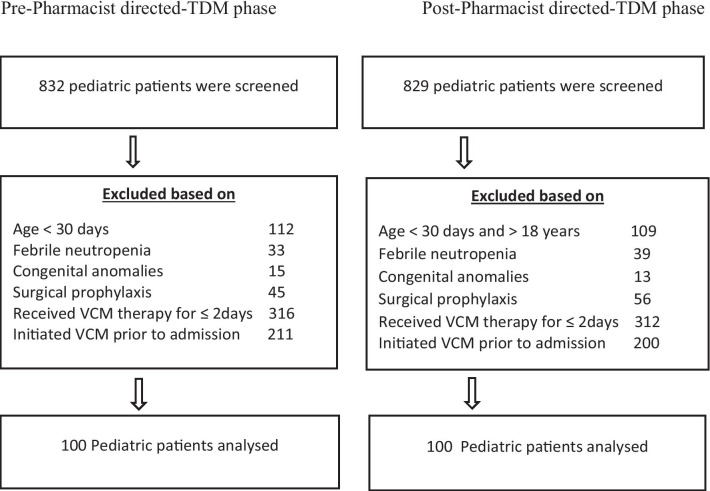
Patient inclusion scheme

In this study, we screened a total of 1650 pediatric patients for eligibility, 756 and 799 cases in the pre–post-implementation phases, respectively, from the 100 cases were enrolled in each phase of the study (Fig. 1). The baseline demographic and clinical characteristics of both groups were comparable. Male participants were higher in both phases. We further divided the patients into five age groups, including Group-I: (1–6 months), Group-II: (6 months–2 years), Group-III: (2–6 years), Group-IV: (6–12 years), and Group-V: (12–18 years). In both phases, the highest proportion of children were of 2–6 years of age (Group-III) who received intravenous vancomycin therapy. A substantial proportion of patients were started vancomycin therapy in the emergency department in both phases (Table 1). A significantly higher number of patients were prescribed optimal initial vancomycin doses in the pharmacist-directed TDM phase compared to the pre-phase of the study (96% vs. 45%). However, from secondary outcomes, we found significant improvement in optimal doses adjustment (50.6% in pre-phase vs. 88.8% in post-phase). Optimal vancomycin levels request improved in post-phase, but it was not statistically significant (37.6% in pre-phase vs. 58.9% in post-phase) (Table 2).

**Table 1:** Baseline demographic and laboratory characteristics of study participants in both phases

Variables	Pre-pharmacist directed-TDM phase (*n* = 100)	Post-pharmacist directed-TDM phase (*n* = 100)	*p* value
Age			
Group-I: children of age 1–6 months	22 (22.0%)	19 (19.0%)	0.895
Group-II: children of > 6 months–2 years	14 (14.0%)	16 (16.0%)	0.887
Group-III: children of > 2–6 years	47 (47.0%)	51 (51.0%)	0.765
Group-IV: children of > 6–12 years	9 (9.0%)	8 (8.0%)	0.998
Group-V: children > 12–18 years	8 (8.0%)	6 (6.0%)	0.958
Sex (male)	61 (61.0%)	59 (59.0%)	0.988
Units of the hospital where VCM therapy initiated			
Emergency	31 (31.0%)	44 (44.0%)	0.075
PICU	11 (11.0%)	10 (10.0%)	0.899
Medicine	21 (21.0%)	18 (18.0%)	0.682
NICU	19 (19.0%)	16 (16.0%)	0.618
Surgery	18 (18.0%)	12 (12.0%)	0.061
Indications of VCM use			
UTI	9 (9%)	7 (7.0%)	0.895
Meningitis	12 (12.0%)	10 (10.0%)	0.956
Endocarditis	2 (2.0%)	1 (1.0%)	0.978
Skin and soft tissue infections	8 (8.0%)	10 (10.0%)	0.959
Bacteraemia	33 (33.0%)	36 (36.0%)	0.897
Pneumonia	28 (28.0%)	27 (27.0%)	0.964
Osteomyelitis	3 (3.0%)	2 (2.0%)	0.785
Intra-abdominal infections	5 (5.0%)	7 (7.0%)	0.823
Baseline laboratory values at VCM initiation			
Creatinine clearance (ml/min)^a^	72.5 (15.5–98.5)	68.9 (10.8–97.2)	0.391
While blood counts (× 10^9^ Cells/L)	10.2 (6.9–15.9)	10.8 (7.1–16.0)	0.699
Patients with impaired renal function	19 (%)	22 (%)	0.119

Data presented as *n* (%) or Median; IQR. Abbreviations: TDM, therapeutic drug monitoring; VCM, vancomycin; PICU, pediatric intensive care unit; NICU, neonatal intensive care unit; UTI, urinary tract infection; ^a^calculation based on Schwartz equation formula

**Table 2:** Comparison of study outcomes in pre and post-implementation phases

Study outcome	Pre-pharmacist directed-TDM phase	Post-pharmacist directed-TDM phase	Mean difference (95% CI)	*p* value
Optimum prescribed initial VCM doses	45.0% (45/100)	96% (96/100)	0.22 (0.14–0.36)	< 0.0001
Optimum VCM-dosage adjustments	50.6% (83/164)	88.8% (95/107)	0.29 (0.15–0.42)	< 0.05
Optimum VCM-sampling time	37.6% (82/218)	58.9% (168/285)	0.15 (− 0.05–0.26)	0.079

**Fig. 2:**
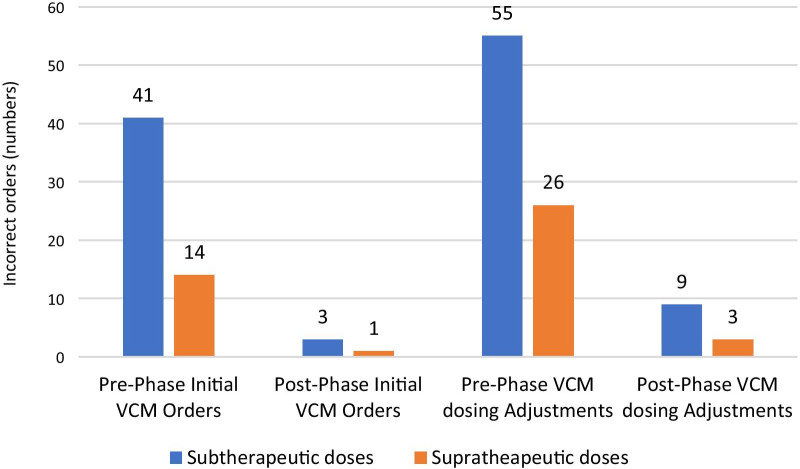
Comparison of subtherapeutic and supratherapeutic VCM starting doses and dosing adjustments in pre and post-implementation phases

**Fig. 3:**
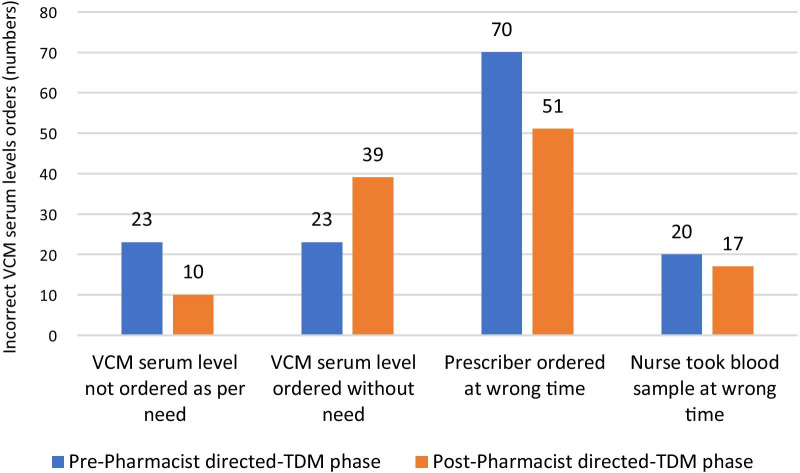
Proportion of incorrect vancomycin serum levels orders

Most of the incorrectly prescribed initial vancomycin doses and adjusted doses were sub-therapeutic in both the study phases (Fig. 2). Data analysis of numbers of incorrectly ordered VCM levels shows that wrong level time prescribed by the physician was the most common reason for incorrect VCM levels (70 in pre-phase vs. 51 in post-phase) (Fig. 3).

The duration of vancomycin therapy was shorter in the post-implementation phase than the pre-implementation phase of the study [median of 8 days; Interquartile range (IQR: 6–15) vs. 11 days; (IQR: 8–16), (*p* = 0.078)].

## Discussion

Therapeutic drug monitoring (TDM) practices are reported to result in the better clinical efficacy of concentration-dependent medications through the improved likelihood of targeted blood levels achievement [14]. Like previous studies, our study also shows the significant benefit of the implementation of pharmacist-directed TDM practices in terms of a higher number of patients dosed with optimal initial vancomycin doses, optimal dosage adjustments in a timely manner, and ordering vancomycin trough levels at the correct time [7, 11]. Comparatively better results are achieved in our study, which might be directly related to the implementation of a collaborative pharmacy practice model instead of isolated efforts.

Alhameed et al. [11] reported significantly improved prescribing of correct initial vancomycin doses through pharmacist-directed TDM services, which raised the bar of correct initial doses from 60 to 91%. However, Marquis et al. [7] studied the pharmacist-led TDM effect on optimal prescribing of vancomycin initial dosing within 24 h and reported a 50% improvement. As a recent study, in the present study, we evaluated entire TDM services as involving optimal initial dose, following dose and frequency adjustments, and vancomycin trough levels, which are important segments of this service to maintain the continuity of care and to attain desired clinical results for patients [11]. Most of the patients prescribed vancomycin need timely achievement of targeted trough levels, which need earlier adjustments of vancomycin dose orders. Therefore, in this study, we considered the prescription order correct if adjusted within 8 h vs. 24 h in the previous study [7]. The selection of a narrow interval for dose adjustments in our study helped us in highlighting and involving inpatient pharmacist importance in TDM processes by their interventions in the absence of clinical pharmacists. Marquis et al. [7] reported 40.4% optimal vancomycin orders within 24 h vs. 75.5% in our study. In addition, Alhameed et al. [11] also selected 8 h’ time interval and reported 60% correct orders within 8 h in their setting. This difference in results can be explained by the consolidated approach of involving all the pharmacists' teams to provide 24/7 TDM services with strong supervision and directions.

The effect of computerized physician order entry (CPOE) had been evaluated in previous studies and reported a moderate effect on vancomycin TDM services [15, 16]. In Damfu et al. [16] study the selected population was only surgical patients, and CPOE operation-oriented educational sessions were conducted for the residents’ teams, responsible for vancomycin order entry. Their study did not involve clinical pharmacists in TDM services. On the other hand, in our setting CPOE is already implemented. In addition, in our study not only the target population was different but also involved the clinical and inpatient pharmacist with dedicated TDM pharmacist. This multidirectional approach in our study resulted in a higher proportion of appropriate initial vancomycin orders. More or less the same results have been reported in another study, where the almost same approach was adopted [11], which shown that the combination of computerized physician order entry use with pharmacist-directed TDM service can be more effective in achieving targeted therapeutic goals.

For the optimal initial dose prescribing, we achieved statistically significant improvement in the pharmacist-directed TDM phase, which we can also correlate with the more focused approach of the pharmacist for appropriateness of the vancomycin doses through careful assessment of all clinical parameters of the patients receiving vancomycin for the first time. Regarding optimal dosage adjustments, we also found statistically significant improvement in the pharmacist-directed TDM-phase compared to the pre-phase. Our results are different than a recently published study, as they reported statistically insignificant improvement in optimal dosage adjustments [11]. Although, Alhameed et al. also adopted the same approach but shared the lack of pharmacist timely follow-up as the major contributing factor for insignificant improvement [11]. However, in our setting pharmacist-directed vancomycin TDM services are performed by the dedicated TDM-pharmacist, clinical pharmacist (doing round with physician team), and inpatient pharmacist (who verify each vancomycin order even for renal adjustments). Top of all the communication among the pharmacist’s team and physicians can play a significant role for the timely dose adjustments.

In terms of optimal vancomycin levels, we found an influential finding that more vancomycin level orders were requested in the pharmacist-directed TDM-phase than in the pre-phase. In addition, we also found that the number of unnecessarily ordered drug levels also increased in the post-phase compared to pre-phase. This is most likely because drug levels orders are placed by the physician, who is more aware of the significance of vancomycin levels but not for the pharmacokinetic-based sampling frequencies and time in pediatric patients [13]. Consequently, leading to additional level orders may be on the request of the pharmacy team to get the accurate blood concentrations. This wastage of resources and extra cost can be curtailed through the physician and nurses’ educational sessions about proper sampling time and frequency for vancomycin during the implementation phase. The empowerment of the pharmacist to place an order for vancomycin levels can be the most cost-effective and efficient option.

This study has few limitations, including; it is a single cantered study of limited study period and generalizability. The quasi-experimental study design has the potential for confounding bias. Although, there was no significant difference in study participants’ demographic and clinical characteristics in both phases. However, variations in unmeasured variables may be present between the groups. Study design associated bias, such as instrumentation and maturation, because of the difference amongst the inpatient pharmacists’ skills in pre and post-phases, which might influence the internal validity. While processing the order in CPOE inpatient pharmacists cannot modify the prescribed medication order unless approved/modified by the physician in CPOE, which might have undervalued the appropriate order recommended by TDM and clinical pharmacists for vancomycin regimen optimization if the prescribing physician did not accept it or respond to inpatient pharmacist’s call. Since drug levels orders are not medication orders, the pharmacist does not get any notification of these orders, therefore, cannot cancel or correct the inappropriately ordered vancomycin levels.

Our study has many strengths. First, participation of a big number of trained pharmacists and subsequent optimal dose adjustments with effective following-ups through robust team communication gave a strong impact on pharmacist-directed TDM services through a collaborative practice model. We were capable of engaging inpatient pharmacists in patient-centered pharmacist-directed clinical TDM services. The quasi-experimental study design is also a big strength of the study, for assessing the practices change in the post-intervention phase. We could study the whole pharmacist-directed TDM services by assessing three main components. To our knowledge, this is the first study in Pakistan to measure the impact of pharmacist-directed VCM therapeutic drug monitoring in the pediatric population.

The focus of future investigations shall be on exploring the impact of the implementation of computerized physician order entry, pharmacist-directed TDM services, and educational intervention of physicians and nurses collectively, to evaluate the results of applying multidepartment collaborative-practice model which provides empowerment to TDM directing pharmacists for complete TDM rights, including drug regimen modifications for optimization and level ordering authority.

## Conclusions

This study establishes the significance of a pharmacist-directed TDM service, which had a positive impact on vancomycin optimal initial dose prescribing and efficient dose adjustments in pediatric patients. However, the result of this study highlights the need for future studies to investigate the opportunities for pharmacy-practices models, which can address the barriers to justified drug levels ordering mechanism in TDM services.

## Data Availability

All data generated or analyzed during this study are included in this published article. The data sets used and/or analyzed during the current study are available from the corresponding author on reasonable request.

## Abbreviations

TDM: Therapeutic drug monitoring
VCM: Vancomycin
ADR: Adverse Drug Effects
PK: Pharmacokinetics
ASHP: American Society of Health-System Pharmacists
PICU: Pediatric Intensive Care Units
NICU: Neonatal Intensive Care Units
PCICU: Pediatric Cardiac Intensive Care Units
AKUH: Aga Khan University Hospital
ERC: Ethical Review Committee
SOPs: Standard Operating Procedures
CHD: Congenital Heart Diseases
IDSA: Infectious Diseases Society of America
MRSA: Methicillin-resistant *Staphylococcus aureus*
